# Evaluation of the neurovascular bundle position at the palate with cone beam computed tomography: an observational study

**DOI:** 10.1186/s13005-015-0097-2

**Published:** 2015-12-30

**Authors:** Hasan Guney Yilmaz, Aysa Ayali

**Affiliations:** Department of Periodontology, Faculty of Dentistry, Near East University, Mersin, Turkey; Department of Oral and Maxillofacial Surgery, Faculty of Dentistry, Near East University, Mersin, Turkey

**Keywords:** CBCT, Palatal artery, Plastic surgery

## Abstract

**Background:**

The aim of this study was to investigate the neurovascular bundle (NVB) position with cone-beam computerized tomography (CBCT).

**Methods:**

CBCT images of 345 patients were evaluated. The distance from the neurovascular bundle to the cemento-enamel junction (CEJ) was measured (DNB). The distance from mid-palatal suture to the alveolar crest was used to determine the palatal depth. Palatal junction angle (PA) was measured using the junction angle between the hard palate and alveolar crest. The relationships between the DNB and the palatal depth and between these two parameters and the PA were evaluated. Student’s *t*-test was used to analyze the differences in DNB related to gender, and the correlation between the DNB while Pearson correlation analysis was used to determine the correlation between the DNB and age (*p* = 0.05). The relationship between the DNB and the palatal depth, and the relationship between these two parameters and the PA were also evaluated using Pearson correlation analysis.

**Results:**

Except at the canine and first premolar areas the DNB was positively correlated with the palatal depth. No significant relationship between the PA and DNB or with PVD was observed. The highest DNB was 14 mm at the first molar, and the lowest was 10.8 mm at the canine.

**Conclusions:**

Care is needed while rotating flap and harvesting the subepithelial connective tissue graft at the canine area because the neurovascular bundle passes approximately 11 mm apically to CEJ at the canine region.

## Background

Palate is the main donor site for subepithelial connective tissue graft (SCTG) at the periodontal plastic surgery [1–3], for pedicle grafts at the oroantral fistula treatments [4] and mucoperiosteal flaps for palatal fistula closure operations [5] in oral and maxillofacial surgery. SCTG and pedicle graft techniques are most common procedures for root coverage [6–9]. Although there is limited gain in the keratinized gingiva tissue thickness when using a pedicle graft, it has an important role over free soft tissue grafts in that the blood supply to the flap is maintained [10]. In contrast, the width of tissue thickness and keratinized gingiva may be increased with free gingival graft and SCTG [2, 3, 10, 11]. The SCTG uses only connective tissue from canine to molar area of the palate as the donor site [12]. In oral and maxillofacial surgery procedures such as oroantral fistula treatment [4], oronasal fistula closure [5] and Lefort 1 osteotomy [13], attention to avoid damaging the neurovascular bundle. If palatal flap technique is used to treat the oroantral fistula, laterally rotated full-partial thickness palatal flap must have a wide base to involve the greater palatine artery (GPA) at the site of its exit from the foramen [4].

Great care is required during harvesting SCTG and rotating the flap from the palate to avoid damaging the neurovascular bundle, which contains the palatal artery, vein, and nerve, to prevent complications such as hemorrhage and paresthesia. The GPA is the major artery supplying the hard palate. The GPA courses anteriorly adjacent to the alveolar ridge, where it then turns upward and leaves the palate superiorly through the foramen incisivus to enter the medial wall of the nasal cavity, terminating and supplying the anterior and inferior nasal septum [11, 14, 15]. The GPA is accompanied by branches of the greater palatine nerve (NVB), which innerves the hard palate and gingiva. Because of these anatomical structures, special attention is required during graft withdrawal from the palate to prevent serious complications. There are a few studies evaluating the position and topography of the NVB by soft tissue dissection of cadaver [15, 16]. However, to the best of our knowledge there are no reports that evaluate the NVB position with CBCT. Therefore the aim of the current study was to investigate the NVB morphology using CBCT images to determine the optimum area for SCTG.

## Methods

The review of patients’ CBCT images was approved by the Ethical Board of the university. The patients which had previous periodontal surgery and current pathology in the palatal area were excluded. Also smokers were excluded from the study. CBCT images of 345 patients (181 male and 164 female) aged from 15 to 69 years (mean 41.1 years) were selected. CBCT (NewTom 3G Quantitative Radiology, Verona, Italy) images were evaluated using a high-definition medical liquid crystal display (LCD) screen (Nio Colar 3MP, Barco, Kortrijk, Belgium) with the program (NNT Viewer, NewTom, Quantitative Radiology, Verona, Italy). Axial, coronal, and sagittal images were obtained with a 1-mm slice thickness.

**Fig. 1:**
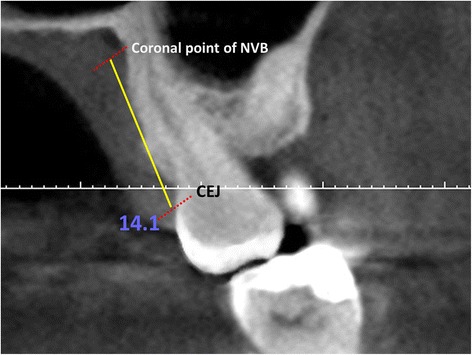
Evaluation of the distance between the NVB and the CEJ

**Fig. 2:**
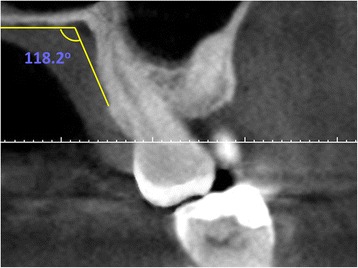
Evaluation of the PA

**Fig. 3:**
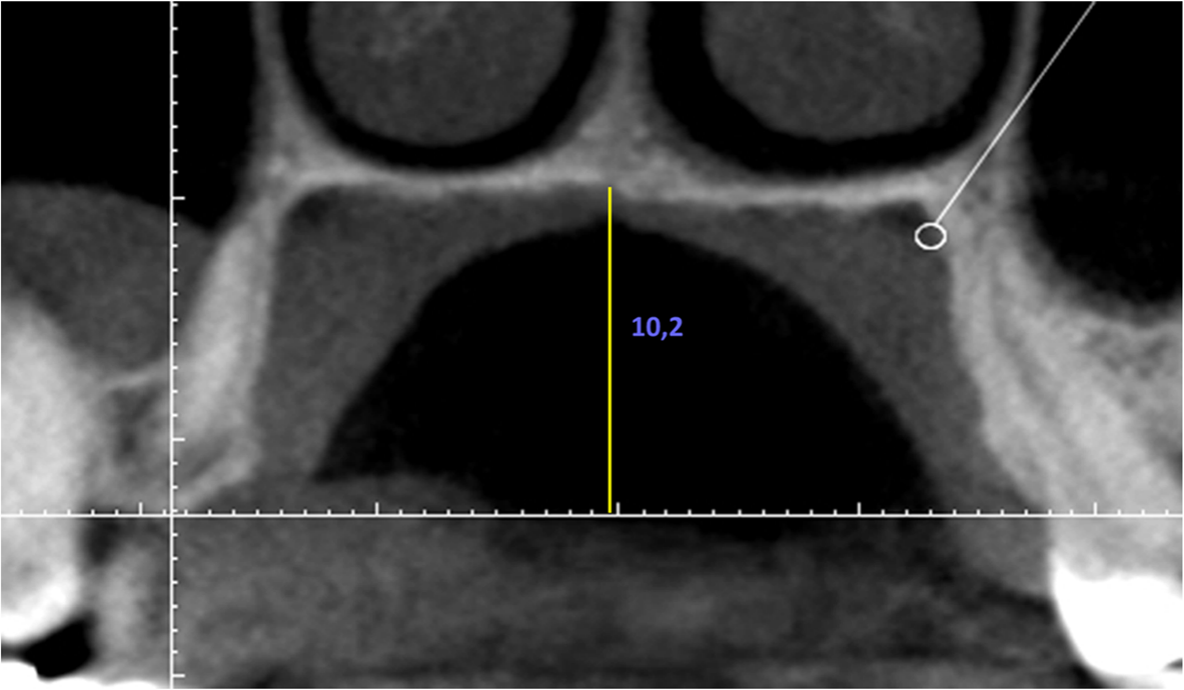
Evaluation of the palatal depth

The distance from the cemento-enamel junction CEJ to the coronal point of the neurovascular bundle was measured from canine to second molar for each tooth (DNB) (Fig. 1). Palatal junction angle (PA) was measured using the junction angle between the hard palate and alveolar crest (Fig. 2). The distance from mid-palatal suture to the alveolar crest was used to determine the palatal depth (Fig. 3). The differences in DNB according to gender and age were evaluated. The relationships between the DNB and the palatal depth and between these two parameters and the PA were evaluated.

Student’s *t*-test was used to analyze the differences in DNB related to gender, and the correlation between the DNB while Pearson correlation analysis was used to determine the correlation between the DNB and age (*p* = 0.05). The relationship between the DNB and the palatal depth, and the relationship between these two parameters and the PA were also evaluated using Pearson correlation analysis.

## Results

The mean and standard deviation values of DNB according to tooth area are shown in Table 1. The DNB was greatest at the first molar region (14.02 mm) and least at the canine site (10.84 mm; Table 1). The DNB and increment of palatal depth were positively correlated primarily at the second molar region (*p* < 0.05; Table 2). There was no correlation between the DNB and PA, and between these parameters and the palatal depth (*p* > 0.05; Table 3). A positive correlation was found between the gender and DNB (*p* = 0.03, *r* = 0.48). There was no correlation between the age and DNB (*p* = 0.24, *r* = 0.18).

**Table 1:** Mean and standard deviation (SD) values of DNB related to the tooth area

DNB	Mean ± SD
M2	13.80 mm ± 1.32
M1	14.02 mm ± 1.44
P2	13.64 mm ± 1.18
P1	11.2 mm ± 2.02
C	10.84 mm ± 2.64

*C* canine; *P1* first premolar; *P2* second premolar; *M1* first molar; *M2* second molar

**Table 2:** Correlation of DNB with PVD

DNB-PVD	r	p
M2	0.61	0.001^*^
M1	0.55	0.001^*^
P2	0.31	0.04^*^
P1	0.23	0.13
C	0.18	0.26

*C* canine; *P1* first premolar; *P2* second premolar; *M1* first molar; *M2* second molar

^*^Statistically significant (*p* < 0.05)

**Table 3:** Correlation of DNB with palatal junction angle (PA)

DNB-PA	r	p
M2	0.04	0.82
M1	0.03	0.72
P2	0.28	0.16
P1	0.09	0.66
C	0.11	0.48

*C* canine; *P1* first premolar; *P2* second premolar; *M1* first molar; *M2* second molar

## Discussion and conclusions

Palatal mucosa is the most common donor site of connective tissue in root coverage surgery and the flap rotating for the treatment of oroantral fistulas. With respect to potential complications of these procedures, the palatine NVB is a highly essential anatomical structure to be protected. Therefore, having a general idea of the possible path of the palatine artery is important. Studies have measured the distance between the coronal border of the NVB path and the alveolar crest [11] or the CEJ to determine the maximum height of the obtained graft [15, 17, 18]. Reiser et al. [17] classified the palatal depth related to NB position as high, average, or shallow in a study using cadavers. The authors reported that the palatal vaults were 7, 12, and 17 mm, respectively, and more tissue could be obtained in areas with a high palatal vault because the neurovascular structures were located distantly from the CEJ. Monnet-Corti et al. [19] measured the length of the palatal vault of 198 maxillary plaster models obtained from consecutive patients. All measurements made with a Boley gauge from canine tooth to the second molar. The results were 12, 13.8, 15.3, 15.1 and 14.7 mm respectively. Similar to the current study, previous studies using cadavers [11, 15, 18] reported that a soft tissue graft can be obtained from the area between maxillary second molar and first premolar region. When the relationship between palatal vault and DNB was evaluated in the present study, authors reported that, this distance showed positive correlation with palatal vault height except first premolar region. In consistent with these results cadaver studies [11, 15] reported that the GPA had the most numerous branches at the first premolar level and that the NVB coursed closely to the alveolar bone crest anteriorly. This outcome indicates that special attention should be warranted at the first premolar region during soft tissue graft withdrawal and incision should not exceed the first premolar region. In another cadaver study, the GPA was examined in 24 skulls to determine the course and the branching pattern of the GPA. The distance from lateral branch of the GPA to the CEJ were 9, 11.1, 13.5, 13.7 and 13.9 mm respectively from canine to second molar and these measurements are in consistent with current study [16]. Kim et al. [12] reported that the distance from CEJ to GPA is 10.6, 12.2, 14, 13.2 and 13.8 mm respectively from canine to second molar tooth in the study that they evaluated the topography of the GPA and the palatal depth using 43 hemisectioned hard palates from 22 adult cadavers. In a recent cadaver study Benninger et al. [20] dissected 17 palates (17 left and right halves) and used electronic digital calipers and a periodontal probe to measure the distance from CEJ to GPA. They reported that the measurements taken with the periodontal probe from CEJ of the maxillary first molar to the GPA revealed a range of 9 to 16 mm (average of 12 mm). And they obtained similar measurements using the digital calipers with a range of 8.9 to 16.1 mm (average of 11.9 mm).

Gingiva, tooth and the other periodontal tissues may be imagined by CBCT and CT as an alternate to existing methods [18, 21–25]. Song et al. [21] performed posterior palatal mucosa thickness measurements using CT of 100 adult patients who underwent computerized tomography on the maxilla for implant surgery. Barriviera et al. [18] used CBCT images to determine the thickness of the palatal mucosa in 31 patients. The aspect ratio of CBCT and CT images is 1:1, and these images can be saved and printed, and multiple measurements can be performed on the computer screen or on hard copies [18]. CBCT presents many advantages like better image quality, lower radiation, lower cost and more patient comfort when compared to conventional tomography [18]. Therefore, in this study, DNB was investigated by CBCT. However, in some cases, distinguishing between the different soft tissues may be impossible on CBCT images. Thus, when performing CBCT, soft tissue retraction (lips and cheeks) is required to distinguish the palatal or buccal mucosa, and the cheek or lip [26]. When compared with CBCT, CT systems demonstrate superior contrast detectability. The limited contrast resolution of CBCT technology makes it difficult to detect different soft tissue structures. Therefore, to extinguish this limitation high definition medical LCD screens were used in the current study.

Great care should be exercised to avoid complications during SCTG harvesting and flap rotation procedures from the palate because the neurovascular bundle passes approximately 11 mm apically to CEJ at the canine region.
